# Access to quality contraceptive counselling among adolescent girls and young women in sub-Saharan Africa

**DOI:** 10.1186/s40834-024-00267-x

**Published:** 2024-04-15

**Authors:** Aliu Mohammed, Irene Esi Donkoh, Richard Gyan Aboagye, Bright Opoku Ahinkorah, Abdul-Aziz Seidu

**Affiliations:** 1 Department of Health, Physical Education and Recreation, University of Cape Coast, Cape Coast, Ghana; 2 Department of Medical Laboratory Science, University of Cape Coast, Cape Coast, Ghana; 3 School of Population Health, Faculty of Medicine and Health, University of New South Wales, Sydney, NSW 2052 Australia; 4 Department of Family and Community Health, Fred N. Binka School of Public Health, University of Health and Allied Sciences, Hohoe, Ghana; 5 School of Clinical Medicine, University of New South Sydney, Sydney, Australia; 6 REMS Consultancy Services Limited, Sekondi-Takoradi, Western Region Ghana; 7 Centre for Gender and Advocacy, Takoradi Technical University, Takoradi, Ghana; 8 College of Public Health, Medical and Veterinary Sciences, James Cook University, Townsville, Australia

**Keywords:** Quality, Contraceptives, Counselling, Sub-Sahara Africa

## Abstract

**Background:**

Improving women’s access to and use of modern contraceptives is a key global strategy for improving the sexual and reproductive health of women. However, the use of modern contraceptives among adolescent girls and young women in sub-Saharan Africa (SSA) remains relatively low, despite the numerous interventions to increase patronage. This study examined adolescent girls and young women’s receipt of quality contraceptive counselling and its associated factors in SSA.

**Methods:**

Data for the study were extracted from the recent Demographic and Health Surveys of 20 countries in SSA, spanning from 2015 to 2021. A sample of 19,398 adolescent girls and young women aged 15 to 24 years was included in the study. We presented the proportion of adolescent girls and young women who received quality contraceptive counselling using a spatial map. Multilevel binary logistic regression analysis was carried out to examine the factors associated with the receipt of quality contraceptive counselling.

**Results:**

Overall, 33.2% of adolescent girls and young women had access to quality contraceptive counselling, ranging from 13.0% in Cameroon to 67.0% in Sierra Leone. The odds of receiving quality contraceptive counselling was higher among adolescent girls and young women aged 20–24 (AOR = 1.48, CI: 1.32–1.67), those with primary (AOR = 1.32, CI: 1.11–1.57) and secondary or higher education (AOR = 1.31, CI: 1.09–1.58), and those married (AOR = 1.32, CI: 1.15–1.52), cohabiting (AOR = 1.47, CI: 1.23–1.76), and previously married (AOR = 1.48, CI: 1.20–1.83) compared to their counterparts in the reference groups. Adolescent girls and young women who were currently working (AOR = 1.22, CI: 1.09–1.37), those who heard of family planning from radio in the last few months (AOR = 1.34, CI:1.21–1.50), those who visited the health facility in the last 12 months (AOR = 1.69, CI: 1.52–1.88), and those residing in the Southern (AOR = 5.01, CI: 3.86–6.51), Eastern (AOR = 2.54, CI: 1.96–3.30), and Western (AOR = 4.09, CI: 3.19–5.25) SSA were more likely to receive quality contraceptive counselling compared to their counterparts in the reference groups. Conversely, adolescent girls and young women who used the internet, those who had problem getting permission to seek medical help, those facing problem in seeking medical help for not wanting to go alone, those from the middle and richest wealth indices, and those from the rural areas were less likely to receive quality contraceptive counselling compared to their counterparts in the reference groups.

**Conclusion:**

Receipt of quality contraceptive counselling among adolescent girls and young women was low. Considering the importance of quality contraceptive counselling on the uptake and continuation of contraception, policymakers need to institute measures that improve adolescent girls and young women’s access to quality contraceptive counselling in SSA, especially in countries like Cameroon, Angola, Madagascar, Mauritania, and Guinea, taking into consideration the factors identified in the study. Increasing adolescent girls and young women’s access to quality contraceptive counselling could greatly minimize the risk of unintended pregnancies and its associated maternal and child health burden in SSA and subsequently contribute to the attainment of the Sustainable Development Goal 3, target 3.7.

## Introduction

Generally, improving women’s access to and use of modern contraceptives remains a major public health strategy for reducing the burden of sexual and reproductive health problems [1, 2]. As such, the Sustainable Development Goal (SDG) 3, target 3.7 emphasizes the need to increase access and use of modern contraceptives among women of reproductive age (15–49 years) worldwide, with adolescent girls and young women aged 15–24 constituting an important aspect [1]. However, despite numerous interventions aimed at improving access to and use of modern contraceptives, the unmet need for contraception among women in sub-Saharan Africa (SSA) remains high [2]. Poor access to quality contraceptives may contribute to low contraception uptake and early discontinuation among women in SSA [3]. Therefore, quality contraceptive counselling remains one of the most viable approaches to improving the use of modern contraceptives among adolescent girls and young women in SSA [4].

Most high-income countries are observing a surge in the prevalence of contraceptive use, providing them an advantage in their quest to achieve SDG 3.7, possibly through the acquisition of knowledge and the provision of access to reliable information, suitable family planning services, and counselling [5, 6]. Evidence from a study conducted in 23 countries in Latin America and the Caribbean indicates that significant progress has been made towards boosting the use of contraceptives [7]. Major attempts are made with regular surveys to assess their success despite some existing gaps, such as improving contraceptive method choice and the use of modern contraceptives to meet family planning needs [8, 9]. With the advancement in the accessibility of high-quality contraceptive information and counseling, individuals now have the ability to decide on the number and spacing of their children. This is achievable through family planning, which also has numerous positive impacts on women’s education and empowerment [7, 10].

However, the rates of inadequate family planning and lack of information on contraceptives are still prevalent in low-and middle-income countries, where one in four women of childbearing age are at risk of unwanted pregnancies or unable to delay childbirth [11]. Although 218 million women of reproductive age in low- and middle-income countries lacked access to contraception in 2019, addressing this issue could significantly reduce the number of unplanned pregnancies, illegal abortions, and maternal deaths from 299,000 to 113,000 annually [10]. Decreasing the unmet need for contraception can be achieved by selecting a family planning method that meets their needs and expectations, minimizes side effects, and is consistently used, or by considering alternative options [8, 12]. In SSA, there is currently a public health crisis as many contraceptive users either stop using their chosen methods or fail to use them correctly due to inadequate contraceptive counselling [13, 14].

There is a varied prevalence of informed contraceptive method choices across the globe [15]. Despite government objectives, a study conducted in 32 countries in SSA demonstrates that the prevalence of informed contraceptive method choice is low, with substantial country variations [16]. The low level of knowledge about contraceptive method choice in SSA is influenced by various factors, such as maternal age, residency, financial status, media exposure, internet use, marital status, education, occupational status, access to health facility, and access to contraceptives [17–19]. This misperception about contraceptives is a significant public health issue, and it affects the prevalence of contraceptive access and use in SSA, which remains unacceptably low [20].

Access to quality contraceptive counselling in SSA is crucial for improving the health and wellbeing of adolescent girls and young women. It also helps to emphasize the importance of educating women to increase their use of modern contraceptives [20, 21]. Modern contraceptives have several advantages, such as protecting against unwanted pregnancies and mortality from unsafe abortions, both of which are prevalent issues in SSA [21, 22]. Given that SSA has a 22.0% prevalence of modern contraceptive use [20], it is essential to ensure that contraceptive counselling is readily available to women of reproductive age.

Despite the availability of resources in SSA like youth-friendly services that provide information on development, unsafe abortion, gender-based violence, sexuality, and family planning, they are not readily accessible [23, 24]. This study sought to examine adolescent girls and young women’s receipt of quality contraceptive information and its associated in SSA. Findings from this study would extend evidence on the need to improve access to quality contraceptive counselling among adolescent girls and young women.

## Methods

### Data source

Data for the study were pooled from the recent Demographic and Health Surveys (DHS) of 20 countries in SSA, spanning from 2015 to 2021. Table 1 contains the list of countries included in the study. We extracted the data from the individual recode files of each of the 20 countries, which can be accessed from the DHS Program [25]. Within the stipulated timeframe (2015–2021), only 20 countries had data on the variables of interest. DHS is a nationally representative survey that has been conducted in over 90 low-and middle-income countries worldwide since its inception [26]. The DHS employed a cross-sectional design with respondents sampled using a two-stage cluster sampling technique. Detailed explanation of the survey methodology can be found in the literature [27, 28]. DHS utilised well-validated questionnaires to collect data from the respondents, with trained data collectors administering the questionnaire. We included a sample of 19,398 adolescent girls (15–19 years) and young women (20–24 years) in the study. This paper was written following the Strengthening Reporting of Observational Studies in Epidemiology (STROBE) guidelines [29].

**Table 1:** Sample distribution per country

Country	Survey year	Pooled weighted sample	Pooled weighted percentage
1. Angola	2015-16	1,106	5.7
2. Benin	2017-18	974	5.0
3. Burundi	2016-17	1,059	5.5
4. Cameroon	2018	857	4.4
5. Ethiopia	2016	1,026	5.3
6. Gambia	2019-20	699	3.6
7. Guinea	2018	697	3.6
8. Liberia	2019-20	427	2.2
9. Madagascar	2021	1,187	6.1
10. Mali	2018	711	3.7
11. Mauritania	2019-21	926	4.8
12. Malawi	2015-16	1,493	7.7
13. Nigeria	2018	2,450	12.6
14. Rwanda	2019-20	891	4.6
15. Sierra Leone	2019	927	4.8
16. Tanzania	2015-16	905	4.7
17. Uganda	2016	1,155	5.9
18. South Africa	2016	507	2.6
19. Zambia	2018	830	4.3
20. Zimbabwe	2015	571	2.9
**All countries**	**2015–2021**	**19,398**	**100.0**

### Variables

Receipt of quality contraceptive counselling was the outcome variable. This variable was measured as a composite variable from three questions, which form the basis of the Method Information Index in quality counselling [18, 30, 31]. In the DHS, women were asked to indicate whether they were told about side effects, how to deal with side effects, and other family planning methods during their family planning counselling services. The response options were “no” and “yes”. We created an index using the responses from the three indicators, with a score of three (3) showing that the respondent received quality contraceptive counselling whilst a score of two (2) or less demonstrates no quality counselling [30, 31]. We coded the quality contraceptive counselling as “1 = yes” and no quality counselling as “0 = no” in the final analysis.

Twenty explanatory variables were included in the study. We selected these variables for inclusion in the study based on their influence on the receipt of quality family planning or contraceptive counselling from the review of pertinent literature [18, 30–33] and their availability in the DHS dataset. The variables consisted of the age of the adolescent girls and young women, level of education, marital status, current working status, watch television, listen to radio, read newspapers or magazines, heard of family planning from newspaper or magazine last few months, heard of family planning from the radio last few months, heard of family planning from television last few months. Others were internet usage, getting medical help for self: permission to go, getting medical help for self: distance to health facility, getting medical help for self: getting money for treatment, getting medical help for self: not wanting to go alone, visit to health facility in the last 12 months, sex of household head, household wealth index, place of residence, and geographical sub-region. For geographical sub-region, all the 20 countries included in the study were grouped into Southern SSA (South Africa, Zambia, and Zimbabwe), Central SSA (Angola and Cameroon), Eastern SSA (Burundi, Ethiopia, Madagascar, Malawi, Rwanda, Uganda, and Tanzania), and Western SSA (Benin, Gambia, Guinea, Liberia, Mali, Mauritania, Nigeria, and Sierra Leone). The last four variables were grouped as contextual level variables (household and community level variables) whilst the remaining variables were termed as individual-level variables.

### Statistical analyses

Data analyses were conducted using Stata software, version 17.0 (Stata Corporation, College Station, TX, USA). We used a spatial map to present the proportion of adolescent girls and young women who received quality contraceptive counselling. Next, we performed a bivariate analysis of the distribution of quality contraceptive counselling across the explanatory variables and used chi-square test of to examine the independent associations between the explanatory and outcome variables. All the variables with p-values < 0.05 from the chi-square test were considered statistically significant and placed in a regression model. We utilised a multilevel binary logistic regression analysis to examine the factors associated with the receipt of quality contraceptive counselling. Four models (Model 0-III) were developed. Model 0 was the model with no explanatory variable, and the results showed the variation in the outcome variable, attributed to the primary sampling unit. Model I, Model II, and Model III included the individual level, contextual level, and all explanatory variables, respectively. Model 0 had random effects results while Models I, II, and III included fixed and random effects results. The fixed effect results showed the association between the explanatory variables and the outcome variable. The results were presented using adjusted odds ratio (AOR) with their respective 95% confidence intervals (CIs). The random effects results denoted the measure of variation in the outcome variable based on the primary sampling unit (measured by Intra-Cluster Correlation Coefficient [ICC]). The Akaike Information Criterion (AIC) and the log-likelihood were used to check for model fitness, or how well different models matched the data. The model with the least AIC and highest log-likelihood was chosen as the best-fitted model for the study and its results were interpreted and discussed. The multilevel regression analysis was conducted using the “melogit” command in Stata. Statistical significance was set at *p* < 0.05. All the analyses were weighted to adjust for disproportionate sampling and the svyset command was employed to cater for the complex nature of the DHS data.

### Ethical consideration

This study utilised publicly available secondary data from the DHS Program. Hence, ethical clearance was not sought for the study. We obtained permission to use the dataset from the Monitoring and Evaluation to Assess and Use Results Demographic and Health Surveys (MEASURE DHS). The detailed ethical issues regarding the DHS are freely available at http://goo.gl/ny8T6X.

## Results

### Receipt of quality contraceptive counselling

**Fig. 1:**
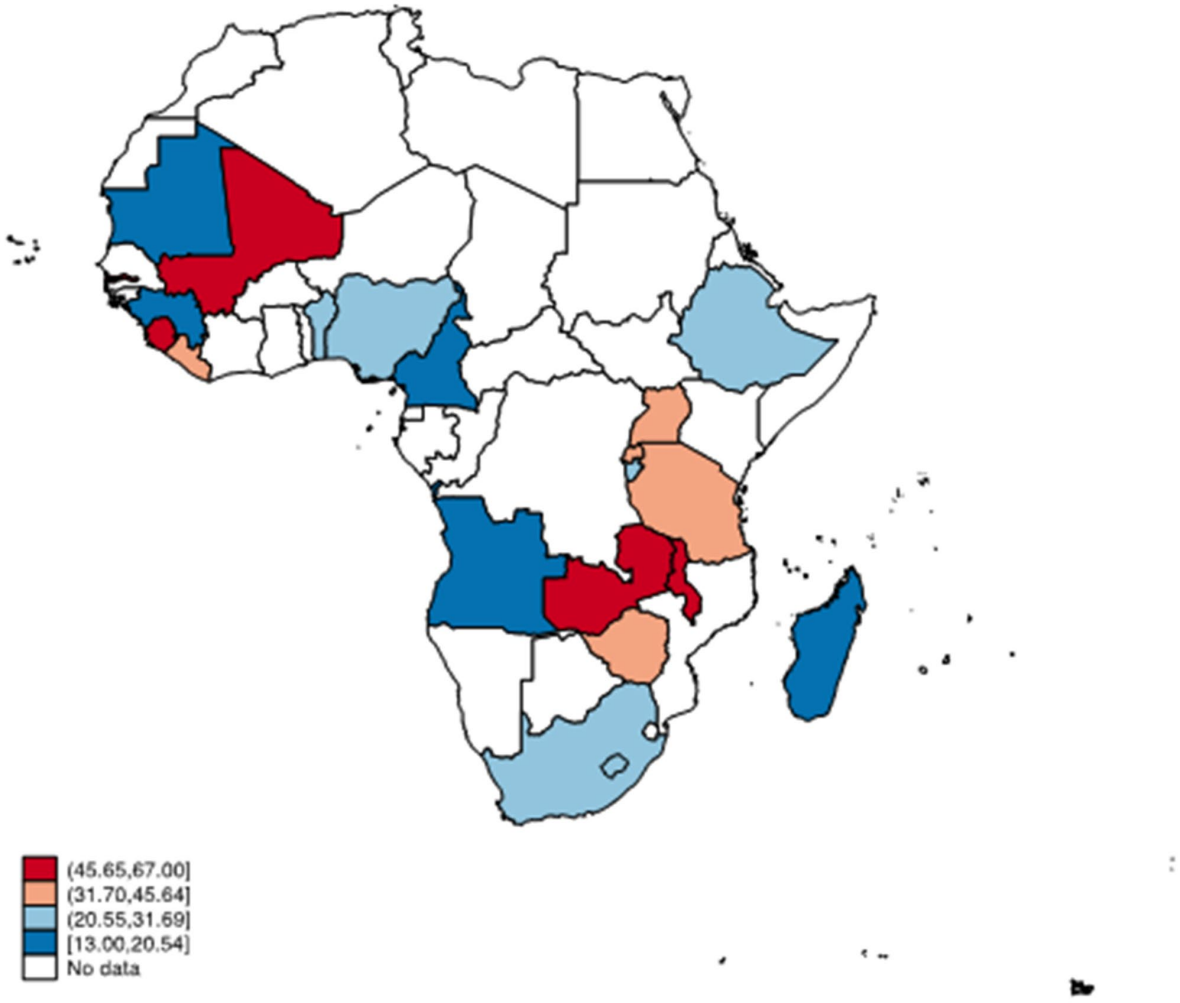
Proportion of adolescent girls and young women who received quality contraceptive counselling

Overall, 33.2% [32.1, 34.2] of adolescent girls and young women received quality contraceptive counselling (Table 2), which ranges from 13.0% in Cameroon to 67.0% in Sierra Leone. Aside from Sierra Leone, more than half of adolescent girls and young women in Gambia (51.8%), Malawi (59.5%), and Zambia (60.3%) received quality contraceptive counselling. On the other hand, less than 20% of adolescent girls and young women in Cameroon (13%), Angola (14.7%), Madagascar (15.7%), Mauritania (16.3%), and Guinea (19.5%) received quality contraceptive counselling (Fig. 1).

**Table 2:** Bivariate results of receipt of quality contraceptive accross the explanatory variables

Variable	Weighted n (%)	Received quality contraceptive counselling		
		No [95% CI] 66.8% [65.8, 67.9]	Yes [95% CI] 33.2% [32.1,34.2]	p-value
**Age (years)**				<0.001
15–19	5,665 (29.2)	75.1 [73.4, 76.6]	24.9 [23.4, 26.6]	
20–24	13,733 (70.8)	63.5 [62.2, 64.7]	36.5 [35.3, 37.8]	
**Level of education**				<0.001
No education	2,428 (12.5)	67.3 [64.1, 70.3]	32.7 [29.7, 35.9]	
Primary	6,541 (33.7)	62.9 [61.2, 64.5]	37.1 [35.5, 38.8]	
Secondary or higher	10,429 (53.8)	69.2 [67.7, 70.7]	30.8 [29.3, 32.3]	
**Marital status**				<0.001
Never in union	7,134 (36.8)	76.0 [74.4, 77.5]	24.0 [22.5, 25.6]	
Married	8,499 (43.8)	61.0 [59.2, 62.7]	39.0 [37.3, 40.8]	
Cohabiting	2,721 (14.0)	63.0 [60.4, 65.5]	37.0 [34.5, 39.6]	
Previously married	1,044 (5.4)	62.3 [58.6, 65.9]	37.7 [34.1, 41.4]	
**Current working status**				<0.001
Not working	8,901 (45.9)	69.8 [68.2, 71.2]	30.2 [28.8, 31.8]	
Working	10,497 (54.1)	64.4 [62.9, 65.8]	35.6 [34.2, 37.1]	
**Watch television**				<0.001
No	9,447 (48.7)	63.5 [62.1, 64.9]	36.5 [35.1, 37.9]	
Yes	9,951 (51.3)	70.0 [68.5, 71.5]	30.0 [28.5, 31.5]	
**Listens to radio**				0.763
No	7,586 (39.1)	67.0 [65.4, 68.6]	33.0 [31.4, 34.6]	
Yes	11,812 (60.9)	66.7 [65.4, 68.1]	33.3 [31.9, 34.6]	
**Reads newspaper or magazine**				<0.001
No	14,741 (76.0)	65.9 [64.6, 67.1]	34.1 [32.9, 35.4]	
Yes	4,657 (24.0)	70.0 [68.1, 71.8]	30.0 [28.2, 31.9]	
**Heard of family planning from newspaper or magazine last few months**				0.118
No	17,467 (90.0)	66.6 [65.4, 67.7]	33.4 [32.3, 34.6]	
Yes	1,931 (10.0)	69.1 [66.1, 72.0]	30.9 [28.0, 33.9]	
**Heard of family planning from radio last few months**				<0.001
No	11,887 (61.3)	68.9 [67.6, 70.2]	31.1 [29.8, 32.4]	
Yes	7,511 (38.7)	63.5 [61.9, 65.2]	36.5 [34.8, 38.1]	
**Heard of family planning from television last few months**				0.068
No	15,347 (79.1)	66.3 [65.2, 67.5]	33.7 [32.5, 34.8]	
Yes	4,051 (20.9)	68.7 [66.4, 71.0]	31.3 [29.0, 33.6]	
**Usage of internet**				<0.001
No	14,293 (73.7)	64.4 [63.2, 65.6]	35.6 [34.4, 36.8]	
Yes	5,105 (26.3)	73.7 [71.6, 75.7]	26.3 [24.3, 28.4]	
**Getting medical help for self: Permission to go**				<0.001
Not a big problem	16,403 (84.6)	65.8 [64.6, 67.0]	34.2 [33.0, 35.4]	
Big problem	2,995 (15.4)	72.5 [70.0, 74.8]	27.5 [25.2, 30.0]	
**Getting medical help for self: Distance to health facility**				0.665
Not a big problem	13,050 (67.3)	66.7 [65.4, 68.0]	33.3 [32.0, 34.6]	
Big problem	6,348 (32.7)	67.2 [65.4, 68.9]	32.8 [31.1, 34.6]	
**Getting medical help for self: Getting money for treatment**				0.001
Not a big problem	10,188 (52.5)	65.3 [63.9, 66.7]	34.7 [33.3, 36.1]	
Big problem	9,210 (47.5)	68.6 [67.1, 70.0]	31.4 [30.0, 32.9]	
**Getting medical help for self: Not wanting to go alone**				<0.001
Not a big problem	15,293 (78.8)	65.7 [64.5, 66.9]	34.3 [33.1, 35.5]	
Big problem	4,105 (21.2)	71.0 [68.9, 73.0]	29.0 [27.0, 31.1]	
**Visited health facility last 12 months**				<0.001
No	7,687 (39.6)	75.1 [73.5, 76.6]	24.9 [23.4, 26.5]	
Yes	11,711 (60.4)	61.4 [60.1, 62.8]	38.6 [37.2, 39.9]	
**Sex of household head**				0.001
Male	14,181 (73.1)	65.9 [64.6, 67.1]	34.1 [32.9, 35.4]	
Female	5,217 (26.9)	69.5 [67.7, 71.3]	30.5 [28.7, 32.3]	
**Wealth index**				<0.001
Poorest	2,579 (13.3)	61.1 [58.5, 63.6]	38.9 [36.4, 41.5]	
Poorer	3,302 (17.0)	63.6 [61.3, 65.9]	36.4 [34.1, 38.7]	
Middle	4,018 (20.7)	67.5 [65.3, 69.7]	32.5 [30.3, 34.7]	
Richer	4,733 (24.4)	67.6 [65.5, 69.7]	32.4 [30.3, 34.5]	
Richest	4,766 (24.6)	70.9 [68.7, 72.9]	29.1 [27.1, 31.3]	
**Place of residence**				<0.001
Urban	8,403 (43.3)	69.1 [67.3, 70.8]	30.9 [29.2, 32.7]	
Rural	10,995 (56.7)	65.1 [63.8, 66.5]	34.9 [33.5, 36.2]	
**Sub-region**				<0.001
Central	1,963 (10.1)	86.1 [83.2, 88.5]	13.9 [11.5, 16.8]	
Southern	1,909 (9.8)	55.7 [53.3, 58.1]	44.3 [41.9, 46.7]	
Eastern	7,716 (39.8)	64.6 [63.2, 66.1]	35.4 [33.9, 36.8]	
Western	7,811 (40.3)	66.9 [64.9, 68.9]	33.1 [31.1, 35.1]	

*p-values were generated from the Pearson chi-square test of independence

### Bivariate results of receipt of contraceptive counselling

Table 2 shows the distribution of quality contraceptive counselling across the explanatory variables in SSA. All the explanatory variables were significantly associated with quality contraceptive counselling among adolescent girls and young women at *p* < 0.05, except listening to radio, heard of family planning from newspapers or magazines in the last few months, heard of family planning from television in the last few months, and have no distance-related problem in getting medical help. A greater proportion of the adolescent girls and young women from the Central part of SSA (86.1%), those never in union (76.0%), those aged 15–19 (75.1%), those who did not visit a health facility in the last 12 months (75.1%), those who use the internet (73.7%), and those having a problem with getting permission to seek medical help (72.5%) received no quality contraceptive counselling.

### Factors associated with receipt of quality contraceptive counselling

Table 3 presents the results of the factors associated with receipt of quality contraceptive counselling among adolescent girls and young women in SSA. With the lowest AIC and highest log-likelihood values, Model III was considered the best fitted model and the interpretations of the results were based on that model. The results showed the odds of recieving quality contraceptive counseling was higher among young women aged 20–24 (AOR = 1.48, CI: 1.32–1.67) compared to adolescent girls (15–19). Adolescent girls and young women who had primary (AOR = 1.32, CI: 1.11–1.57) and secondary or higher education (AOR = 1.31, CI: 1.09–1.58) were more likely to receive quality contraceptive counselling compared to those who had no education. Married (AOR = 1.32, CI: 1.15–1.52), cohabiting (AOR = 1.47, CI: 1.23–1.76), and previously married (AOR = 1.48, CI: 1.20–1.83) adolescent girls and young women were more likely to receive quality contraceptive counselling relative to those who had never being in union. The odds of receiving quality contraceptive counselling was higher among currently working adolescent girls and young women compared to those who were not working (AOR = 1.22, CI: 1.09–1.37). Additionally, the odds of receipt of quality contraceptive counselling was higher among adolescent girls and young women who heard of family planning from radio in the last few months (AOR = 1.34, CI:1.21–1.50), visited a health facility in the last 12 months (AOR = 1.69, CI: 1.52–1.88), and those residing in the Southern (AOR = 5.01, CI: 3.86–6.51), Eastern (AOR = 2.54, CI: 1.96–3.30), and Western (AOR = 4.09, CI: 3.19–5.25) parts of SSA. Meanwhile, those who use the internet (AOR = 0.78, CI: 0.67–0.91), had problems getting permission to seek medical help (AOR = 0.83, CI: 0.72–0.97), had problems in seeking medical help for not wanting to go alone (AOR = 0.79, CI: 0.70–0.89), those from the middle (AOR = 0.80, CI: 0.68–0.94) and richest (AOR = 0.80, CI: 0.64–0.99) wealth index, and those in rural areas (AOR = 0.85, CI: 0.73–0.99) were less likely to receive quality contraceptive counselling.

**Table 3:** Factors associated with receipt of quality contraceptive counselling among adolescent girls and young women in sub-Saharan Africa

Variable	Model O Empty model	Model I AOR [95% CI]	Model II AOR [95% CI]	Model III AOR [95% CI]
** Age (years)**				
15–19		1.00		1.00
20–24		1.47^***^ [1.31, 1.65]		1.48^***^ [1.32, 1.67]
**Level of education**				
No education		1.00		1.00
Primary		1.15 [0.97, 1.35]		1.32^**^ [1.11, 1.57]
Secondary or higher		1.19^*^ [1.01, 1.42]		1.31^**^ [1.09, 1.58]
**Marital status**				
Never in union		1.00		1.00
Married		1.42^***^ [1.24, 1.62]		1.32^***^ [1.15, 1.52]
Cohabiting		1.24^**^ [1.06, 1.46]		1.47^***^ [1.23, 1.76]
Previously married		1.36^**^ [1.12, 1.67]		1.48^***^ [1.20, 1.83]
**Current working status**				
Not working		1.00		1.00
Working		1.12^*^ [1.01, 1.25]		1.22^***^ [1.09, 1.37]
**Read newspaper or magazine**				
No		1.00		1.00
Yes		0.86^*^ [0.76, 0.97]		0.91 [0.80, 1.04]
**Watch television**				
No		1.00		1.00
Yes		0.92 [0.82, 1.03]		0.94 [0.84, 1.07]
**Heard of family planning from radio last few months**				
No		1.00		1.00
Yes		1.29^***^ [1.16, 1.43]		1.34^***^ [1.21, 1.50]
**Usage of internet**				
No		1.00		1.00
Yes		0.80^**^ [0.70, 0.92]		0.78^**^ [0.67, 0.91]
**Getting medical help for self: Permission to go**				
Not a big problem		1.00		1.00
Big problem		0.80^**^ [0.69, 0.93]		0.83^*^ [0.72, 0.97]
**Getting medical help for self: Getting money for treatment**				
Not a big problem		1.00		1.00
Big problem		0.96 [0.86, 1.07]		1.02 [0.92, 1.14]
**Getting medical help for self: Not wanting to go alone**				
Not a big problem		1.00		1.00
Big problem		0.76^***^ [0.67, 0.86]		0.79^***^ [0.70, 0.89]
**Visited health facility last 12 months**				
No		1.00		1.00
Yes		1.64^***^ [1.47, 1.82]		1.69^***^ [1.52, 1.88]
**Sex of household head**				
Male			1.00	1.00
Female			0.86^**^ [0.77, 0.95]	0.94 [0.84, 1.06]
**Wealth index**				
Poorest			1.00	1.00
Poorer			0.94 [0.80, 1.10]	0.93 [0.79, 1.09]
Middle			0.82^*^ [0.70, 0.95]	0.80^**^ [0.68, 0.94]
Richer			0.90 [0.76, 1.06]	0.89 [0.75, 1.06]
Richest			0.76^**^ [0.63, 0.93]	0.80^*^ [0.64, 0.99]
**Place of residence**				
Urban			1.00	1.00
Rural			0.90 [0.78, 1.04]	0.85^*^ [0.73, 0.99]
**Sub-region**				
Central			1.00	1.00
Southern			5.73^***^ [4.44, 7.41]	5.01^***^ [3.86, 6.51]
Eastern			3.61^***^ [2.80, 4.65]	2.54^***^ [1.96, 3.30]
Western			4.00^***^ [3.13, 5.13]	4.09^***^ [3.19, 5.25]
**Random effect model**				
PSU variance (95% CI)	6.019 [5.025, 7.212]	5.166 [4.277, 6.238]	6.116 [5.101, 7.335]	5.656 [4.707, 6.796]
ICC	0.647	0.611	0.650	0.632
Wald chi-square	Reference	422.18 (< 0.001)	218.72 (< 0.001)	618.47 (< 0.001)
**Model fitness**				
Log-likelihood	-183281.65	-177142.64	-179032.75	-173,312
AIC	366567.3	354319.3	358087.5	346,676
*N*	19,398	19,398	19,398	19,398
Number of clusters	1,104	1,104	1,104	1,104

aOR = adjusted odds ratios; CI = Confidence Interval; ^*^p < 0.05, ^**^p < 0.01, ^***^p < 0.001; 1.00 = Reference category; PSU = Primary Sampling Unit; ICC = Intra-Cluster Correlation Coefficient; AIC = Akaike Information Criterion

## Discussion

In this study, we found that the proportion of adolescent girls and young women who received quality contraceptive counselling in SSA is low (33.2%), ranging from 13% in Cameroon to 67% in Sierra Leone. Aside from Sierra Leone, only Zambia, Malawi, and Gambia had more than half of their adolescent girls and young women receiving quality contraceptive counselling. Meanwhile, about 80% of the adolescent girls and young women in Cameroon, Angola, Madagascar, Mauritania, and Guinea did not receive quality contraceptive counselling. Further, we found that age, education, marital status, employment, media exposure, healthcare access, and geographic location were significantly associated with receipt of quality contraceptive counselling among adolescent girls and young women.

In concordance with the findings from previous studies [30, 34, 35], we found that most adolescent girls and young women in SSA do not receive quality contraceptive counselling. Perhaps, the ineffective implementation of sexual and reproductive health policy strategies [36] as well as the attitude toward contraceptive counselling [37, 38] might have contributed to the limited access to quality contraceptive counselling among adolescent girls and young women in SSA. Thus, considering the importance of quality contraceptive counselling in reducing the fertility rate [39], evidenced-based country-specific counselling strategies are needed to improve women’s access to quality contraceptive counselling in SSA.

We also observed wide inter-country variations in the proportion of adolescent girls and young women who received quality contraceptive counselling in SSA. Although we could not make a direct comparison due to the non-availability of a similar multi-country level study, a few individual country-level comparisons revealed varied discrepancies. For example, while our finding in Sierra Leone was marginally higher (67%) than what was previously reported [63.4%] [31], we recorded a lower (21.6%) proportion of adolescent girls and young womenȉs receipt of quality contraceptive counselling than previously estimated (30%) in Ethiopia [30]. These observed differences could be partly explained by the variations in study designs, timing, and target population. For example, while Sserwanja et al. [31] studied women aged 15–24 years in Sierra Leone, Hrusa et al. [30] surveyed women of reproductive age (15–49 years) in Ethiopia. Nonetheless, the observed differences in our findings relative to the findings from the previous studies give some credence to the raging concern that while access to quality contraceptive counselling is improving in some countries in SSA, others are witnessing a decline [34]. This highlights the need for concerted efforts to improve women’s access to quality contraceptive counselling in SSA. Lack of access to quality contraceptive counselling has been associated with women’s likelihood not to choose any form of contraceptive [39] as well as a high rate of contraceptive discontinuation [40], which contributes to increased fertility rate in SSA [41].

Although previous studies [30, 31] reported no significant association between women’s age and receipt of quality contraceptive counselling information, our findings revealed that young women aged 20–24 years were more likely to receive quality contraceptive counselling relative to adolescent girls. The observed discrepancies in our findings relative to the previous studies could be due to methodological variations since previous studies were conducted at an individual country level compared to our use of multi-country settings. Meanwhile, among women of reproductive age, Semachew Kasa et al. [42] reported that awareness, attitude, and practices toward family planning increase with advancing age. This might contribute to increased demand or search for information on contraception and thus, increase access to quality contraceptive counselling among women aged 20–24 years. Perhaps, providing adolescent-friendly contraceptive counselling services could significantly improve quality contraceptive counselling information and contraceptive uptake among adolescent girls. Also, concerted efforts should be made in providing comprehensive sexual education to adolescent girls, equipping them with the quality information on contraceptives that might influence its use.

Adolescent girls and young women who were employed (currently working) as well as those having primary, secondary, or higher education were more likely to receive quality contraceptive counselling relative to the unemployed and those with no formal education. Similar findings were reported in previous studies [30, 43]. Education and employment have been identified as major empowerment tools in women’s autonomy and decision-making capacity towards contraception practices [44–46] such as accessing contraceptive counselling. Perhaps, interventions targeted at increasing adolescent girls and young women’s access to quality contraceptive counselling must pay particular attention to those with no education and the unemployed to equip them with the requisite skills and agency to seek health care including contraceptives.

Also, our findings revealed that adolescent girls and young women who were married, cohabiting, or previously married had higher odds of receiving quality contraceptive counselling relative to those who were never in a union. This finding contradicts findings from previous studies in SSA [30, 31] that reported no significant relationship between women’s marital status and their likelihood of receiving quality contraceptive counselling. Meanwhile, available evidence suggests that married and cohabiting women are more interested in the use of contraceptives [47], since they engage more in sexual intercourse relative to those who were never in a union [48]. Perhaps, this may contribute to their increased likelihood of receiving quality contraceptive counselling information.

Further, we found that adolescent girls and young women who heard of family planning from the radio in the last few months were more likely to receive quality contraceptive counselling. A similar finding was reported in a previous study in Ethiopia [43]. The importance of radio in promoting sexual and reproductive health education and practices in SSA has been highlighted in several studies [43, 49, 50]. Local radio stations in SSA commonly engage resource persons to educate the public on important health matters such as contraception [51], which could improve women’s ability to access quality contraceptive counselling.

Interestingly, adolescent girls and young women who use the internet had lower odds of receiving quality contraceptive counselling. Although the internet is a very useful tool for disseminating important health information, it is also a breeding ground for disinformation, particularly on contraception [52]. Given that many women in SSA Africa have limited education, they may have less ability to access quality contraceptive counselling from internet sources. Therefore, strategies to improve adolescent girls and young women’s access to quality contraceptive counselling could include education on responsible usage of internet-based health information. Also, internet-based campaigns can be developed and implemented in SSA to provide quality information on contraceptives.

Further, adolescent girls and young women who had increased or easy access to healthcare services (e.g., those who visited a health facility in the last 12 months or those who had no problem getting permission to seek medical help) were more likely to receive quality contraceptive counselling. Understandably, visiting healthcare facilities increases women’s interaction with trained healthcare staff, which could improve their ability to access quality contraceptive counselling from healthcare workers [39]. Scott et al. [53] reported that increased access to contraceptive and services from healthcare workers significantly improves the use of modern contraception among women. This finding highlights the importance of continuously educating young women on the need to access contraceptive counselling and services from trained healthcare workers.

Previous studies in Ethiopia [30], Sierra Leone [31], and Uganda [35] reported significant geographic variations in women’s receipt of quality contraceptive counselling, albeit at the intra-country level. In this study, we observed high access to quality contraceptive counselling among the young women who resided in the Southern, Eastern, and Western parts of SSA relative to those from Central SSA. Perhaps, the varied socio-economic and cultural differences of the various sub-regions might have contributed to the observed variations. For instance, Lukyamuzi et al. [35] reported that people in geographical locations with increased access to healthcare services often receive quality contraceptive counselling.

We observed that adolescent girls and young women from rural areas had lower odds of receiving quality contraceptive counselling information. This may be attributed to their limited access to healthcare services, including contraceptive counselling services [54]. Limited healthcare infrastructural facilities and personnel could have contributed to the lower likelihood of adolescent girls and young women receiving quality contraceptive counselling. Also, deep-rooted sociocultural beliefs could have negatively affected the adolescent girls and young women’s likelihood of accessing healthcare including quality contraceptive counselling.

### Strength and limitations

Our use of nationally representative datasets to examine receipt of quality contraceptive counselling among adolescent girls and young women across 20 countries in SSA enhances the generalizability of our findings to adolescent girls and young women in SSA. Nonetheless, this study had some limitations. Although our analysis was based on the most recent DHS datasets of the individual countries, the survey years varied. This could limit the interpretation of our findings, particularly when comparing women’s receipt of quality contraceptive counselling information from one country to another. Also, while facility and healthcare worker-related factors could influence women’s access to and receipt of quality contraceptive counselling, our analysis focused on only client-level variables. The DHS survey adopts cross-sectional study design, hence only associations are reported. Additionally, since adolescent girls and young women’s receipt of quality contraceptive counselling was self-reported, it is open to recall and social desirability biases.

## Conclusion

This study highlights the limited access to quality contraceptive counselling among adolescent girls and young women across SSA. Considering the importance of quality contraceptive counselling on the uptake and continuation of contraception, policymakers need to institute measures that improve young women’s access to quality contraceptive counselling in SSA, especially in countries like Cameroon, Angola, Madagascar, Mauritania, and Guinea. Such measures could be targeted at adolescent girls and young women aged 15–19 years, those with no education, those who are single, those unemployed, those with limited access to radio, those with difficulty accessing healthcare, and those from the central part of SSA. Perhaps, increasing adolescent girls and young women’s receipt of quality contraceptive counselling could greatly minimize the risk of unintended pregnancies and its associated maternal and child health burden in SSA.

## Data Availability

The data used for this study is freely available at http://dhsprogram.com/data/available-datasets.cfm.
